# Non-Vitamin K Antagonist Oral Anticoagulants in Patients with Atrial Fibrillation and Valvular Heart Disease

**DOI:** 10.3390/jcm8101624

**Published:** 2019-10-04

**Authors:** Inki Moon, So-Ryoung Lee, Eue-Keun Choi, Euijae Lee, Jin-Hyung Jung, Kyung-Do Han, Myung-Jin Cha, Seil Oh, Gregory Y.H. Lip

**Affiliations:** 1 Department of Internal Medicine, Seoul National University Hospital, Seoul 03080, Korea; rokstone4330@gmail.com (I.M.); minerva1368@gmail.com (S.-R.L.); euijae14@gmail.com (E.L.); cmj.with.love@gmail.com (M.-J.C.); seil@snu.ac.kr (S.O.); 2 Department of Medical Statistics, College of Medicine, Catholic University of Korea, Seoul 06591, Korea; jungjin115@naver.com (J.-H.J.); hkd917@naver.com (K.-D.H.); 3 Liverpool Centre for Cardiovascular Science, University of Liverpool and Liverpool Chest & Heart Hospital, Liverpool L14 3PE, UK; Gregory.Lip@liverpool.ac.uk; 4 Department of Clinical Medicine, Aalborg University, 9000 Aalborg, Denmark

**Keywords:** atrial fibrillation, valvular heart disease, non-vitamin K antagonist oral anticoagulant, stroke, bleeding

## Abstract

Background: There are limited data for non-vitamin K antagonist oral anticoagulants (NOACs) impact on outcomes for patients with atrial fibrillation (AF) and valvular heart diseases (VHDs). Methods: We identified patients with AF and associated Evaluated Heartvalves, Rheumatic or Artificial (EHRA) type 2 VHDs, and who had been naïve from the oral anticoagulants in the Korean National Health Insurance Service database between 2014 and 2016 (warfarin: *n* = 2671; NOAC: *n* = 3058). For analyzing the effect of NOAC on primary prevention, we excluded those with a previous history of ischemic stroke, intracranial hemorrhage (ICH), and gastrointestinal (GI) bleeding events. To balance covariates, we used the propensity score weighting method. Ischemic stroke, ICH, GI bleeding, major bleeding, all-cause death, and their composite outcome and fatal clinical events were evaluated. Results: During a follow-up with a mean duration of 1.4 years, NOACs were associated with lower risks of ischemic stroke (hazard ratio (HR): 0.71, 95% confidence interval (CI): 0.53–0.96), GI bleeding (HR: 0.50, 95% CI: 0.35–0.72), fatal ICH (HR: 0.28, 95% CI: 0.07–0.83), and major bleeding (HR: 0.61, 95% CI: 0.45–0.80) compared with warfarin. Overall, NOACs were associated with a lower risk of the composite outcome (HR: 0.68, 95% CI: 0.58–0.80). Conclusions: In this nationwide Asian AF population with EHRA type 2 VHDs, NOAC use was associated with lower risks of ischemic stroke, major bleeding, all-cause death, and the composite outcome compared to warfarin use.

## 1. Introduction

Atrial fibrillation (AF) often co-exists with many types of valvular heart diseases (VHDs); for example, AF patients with mitral stenosis or mechanical heart valves have a significantly higher risk of thromboembolic events than those with non-valvular AF [1]. Thus, pivotal randomized clinical trials (RCTs) for the evaluation of efficacy and safety of non-vitamin K antagonist oral anticoagulants (NOACs) in AF exclude these with mechanical heart valves and moderate to severe mitral stenosis [2,3,4,5]. In these pivotal RCTs, the effectiveness and safety of NOACs for stroke prevention was demonstrated, and NOACs are now widely used for stroke prevention in the AF population without Evaluated Heartvalves, Rheumatic or Artificial (EHRA) type 1 VHDs [6,7,8,9].

However, the population of these pivotal RCTs includes patients with mitral regurgitation, aortic stenosis, and aortic regurgitation, which are defined as EHRA type 2 VHDs. Additionally, the impact of these EHRA type 2 VHDs for clinical outcomes in patients with AF is controversial [1] and the performance of NOACs compared to that of warfarin in patients with EHRA type 2 VHDs remains questionable. In a recent meta-analysis of these RCTs, the pooled high-dose NOAC group shows a significantly lower risk of thromboembolic events and a similar risk of major bleeding compared with the warfarin group [10]. Subsequently, there are several comparative analyses between NOACs and warfarin in patients with EHRA type 2 VHDs based on real-world data; however, the benefits of NOAC compared to warfarin are inconsistent [11,12]. Furthermore, considering the greater benefit of NOAC regarding risk reduction of major bleeding in Asians compared to that in non-Asians, there are few large-sized datasets focused on Asian patients with EHRA type 2 VHDs [13,14,15].

Given that VHDs commonly co-exist in Asian patients with AF, we aimed to evaluate the effectiveness and safety of NOACs compared to those of warfarin, specifically in AF patients with EHRA type 2 VHDs, using a large real-world nationwide cohort of Korean individuals.

## 2. Methods

### 2.1. Data Source

This study used the national claims data established by the National Health Insurance Service (NHIS) of Korea. The NHIS is the single insurer managed by the Korean government, and the entire Korean population comprises mandatory subscribers. The Korean NHIS database contains the following information of the entire Korean population: Sociodemographic data and all medical expenses for both inpatient and outpatient services, including pharmacy dispensing claims, and mortality data [16,17]. Diagnoses in this database are coded using the International Classification of Disease-10th Revision-Clinical Modification (ICD-10-CM) nomenclature. This study was exempted from the review by the Seoul National University Hospital Institutional Review Board (E-1804-002-932). All data and materials have been made publicly available at the National Health Insurance Sharing Service and can be accessed at https://nhiss.nhis.or.kr/bd/ab/bdaba000eng.do.

### 2.2. Study Cohort

**Figure 1:**
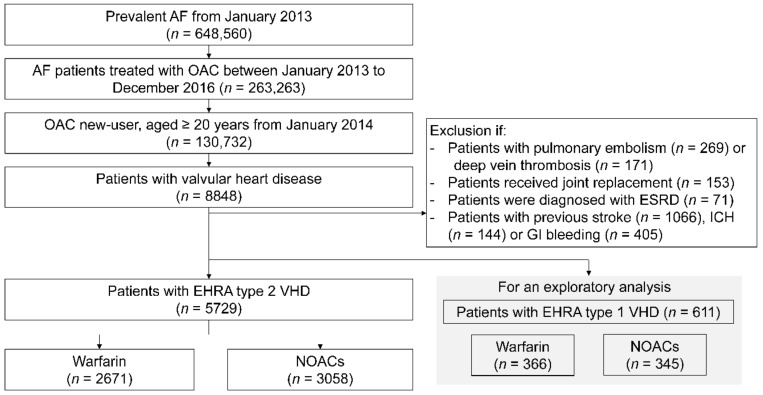
Study population enrollment flow. A total of 5729 patients with EHRA type 2 VHDs were included in the final analysis. We conducted an exploratory analysis for EHRA type 1 VHD patients (patients with “valvular AF”) (*n* = 611). Abbreviations: AF, atrial fibrillation; EHRA, Evaluated Heartvalves, Rheumatic or Artificial; ESRD, end-stage renal disease; GI, gastrointestinal; NOAC, non-vitamin K antagonist oral anticoagulant; OAC, oral anticoagulant, VHD, valvular heart disease.

We included adult patients with AF (ICD-10-CM code I480-484, I489) who were newly prescribed oral anticoagulants (OACs) from January 2014 to December 2016. We also excluded those who had a history of OAC prescriptions between January 2013 and December 2013, so as to include only those who were OAC-naïve. Then, we identified AF patients with VHDs including mitral valve disease (ICD-10-CM code I34.0-2), aortic valve disease (I35.0-2, I06.0-2), tricuspid valve disease (I36.0-2, I07.0-2), and pulmonary valve disease (I37.0-2). Patients with EHRA type 1 VHDs (rheumatic mitral stenosis (I05.0, I05.2, I05.9) or prosthetic heart valve (Z95.2-4)) were selected for a separate analysis. We excluded other alternative OAC treatment indications, such as pulmonary embolism, deep vein thrombosis, or joint replacement surgery. Additionally, patients with end-stage renal disease were excluded. To enroll patients who used OACs only for primary prevention, we excluded those with a previous history of ischemic stroke, intracranial hemorrhage (ICH), and gastrointestinal (GI) bleeding events. The detailed patient enrollment flow is presented in Figure 1.

### 2.3. Defining the Comorbidities and Outcomes

Comorbidities including hypertension, diabetes mellitus, dyslipidemia, congestive heart failure (CHF), history of myocardial infarction (MI), peripheral artery disease (PAD), chronic obstructive pulmonary disease (COPD) were defined by the ICD-10-CM codes, and the detail of definition was shown in Table S1 (Supplementary Materials). The CHA_2_DS_2_-VASc score was calculated for each patient by assigning 1 point each for the age interval between 65 and 74 years, female sex, the presence of hypertension, diabetes mellitus, CHF, and presence of vascular disease (prior MI or PAD) and 2 points each for a history of stroke/transient ischemic attack/systemic thromboembolism and the age of ≥75 years [18].

We evaluated 6 clinical outcomes to analyze the effectiveness and safety of NOAC versus warfarin [14,19]. Ischemic stroke, ICH, hospitalization for GI bleeding, hospitalization for major bleeding (ICH + GI bleeding), all-cause death, and the “composite outcome” (ischemic stroke + major bleeding (ICH + hospitalization for GI bleeding) + all-cause death) were defined by ICD-10-CM codes. Full details are summarized in Table S2 (Supplementary Materials). To evaluate the severity of clinical events, fatal ischemic stroke, fatal ICH, fatal GI bleeding, fatal major bleeding, and the composite of these fatal events were also analyzed. Any event that led to death within 30 days of its occurrence was defined as a fatal event. Patient follow-up started from the initiation of index treatment and ended with the occurrence of the clinical outcome events or the end of study period (31 December 2016), whichever came first.

### 2.4. Subgroup Analyses

Subgroup analyses were performed based on patients’ baseline characteristics including age, sex, CHA_2_DS_2_-VASc score, and type of VHD. In the age subgroup analysis, we categorized patients into 3 groups as follows: Ages of <65, 65–74, and ≥75 years old. Regarding the CHA_2_DS_2_-VASc score, we divided the total study population into two groups by 0 to 2 and 3 or more points. In the type of VHD, we divided patients who presented mitral regurgitation or other VHDs.

### 2.5. Statistical Analysis

We used the inverse probability of treatment weighting (IPTW) method to reduce the impact of treatment-selection bias and the potential confounding factors between the two treatment groups. IPTW used the whole dataset and assigned an inverse probability of received treatment weighting by applying 1/propensity score (PS) to patients in the treated cohort and (1/(1 – PS)) to those in the control cohort. The propensity for each treatment group was estimated using a logistic regression method including all clinical variables: Age, sex, CHA2DS2-VASc score, hypertension, diabetes mellitus, dyslipidemia, CHF, prior MI, COPD, and PAD (Table S3, Supplementary Materials). After calculating the stabilized weights from propensity scores, IPTW effectively generated a pseudo-population with the near-perfect-covariate balance between treatment groups [20]. To reduce the effect of extremely small and large weights, we trimmed at the 5th and 95th percentiles of the weights. The balance of each covariate between two treatment groups was examined by the absolute standardized difference (ASD). An ASD of 0.1 or less was considered to balance each covariate between the two treatment groups [21].

The comparison of cumulative incidence rates between the NOAC and warfarin groups was performed based on Kaplan–Meier censoring estimates using the log-rank test. Incidence rates were described as the number of events per 100 person-years (PY). The risk for 6 clinical outcomes and fatal events of the two treatment groups were presented with hazard ratios (HRs), and the corresponding 95% confidence intervals (CIs) calculated by applying weighted Cox proportional hazard models with IPTW. For subgroup analyses, we used a multivariable Cox proportional hazard regression method adjusting for age, sex, CHA_2_DS_2_-VASc score, hypertension, diabetes mellitus, dyslipidemia, heart failure, prior MI, PAD, and COPD. All *P*-values were two-sided, and a value lower than 0.05 was considered statistically significant. Statistical analyses were performed using SAS version 9.3 (SAS Institute, Cary, NC, USA).

### 2.6. Sensitivity Analyses

A sensitivity analysis was performed with the multivariable Cox proportional hazard regression method using all variables including PS calculation and socioeconomic status. Additionally, to adjust the difference of follow-up duration between NOAC and warfarin groups, we performed sensitivity analyses with the restriction of the follow-up period to 6 months and 1 year [19].

To adjust for possible confounding factors, we performed multivariable Cox proportional hazard regression with chronic kidney disease (CKD), chronic hepatitis, liver cirrhosis, and antiplatelets. CKD (N18), chronic hepatitis (B15–19), liver cirrhosis (K70–77) were defined using ICD-10-CM codes. The use of antiplatelets was defined as a prescription of any antiplatelet agent (aspirin or clopidogrel). In this study, the primary analysis was conducted in analogy with the intention-to-treat principle, regardless of subsequent treatment changes. We performed a sensitivity analysis similar to the on-treatment principle [22]. In the on-treatment analysis, patients who discontinued the index treatment during the study period were censored. Discontinuation of index treatment was defined as the intervals of 30 days from the last day of the last drug supply.

## 3. Results

### 3.1. Baseline Characteristics of the Study Population

We identified 5729 patients as new users of OAC treatment with EHRA type 2 VHDs (Figure 1). The study population was distributed according to the OAC type: Warfarin (*n* = 2671, 47%) and NOAC (*n* = 3058, 53%). Among patients on NOAC, 24.7% (*n* = 756) of patients received dabigatran, 41.9% (*n* = 1281) received rivaroxaban, 25.3% (*n* = 774) received apixaban, and 8.1% (*n* = 247) received edoxaban. The average follow-up duration was 1.3 years.

Table 1 presents the baseline characteristics of the study population before and after IPTW. Before weighting, patients with NOAC were older (mean age: 73.5 ± 9.2 vs. 66.5 ± 13.2 years for NOAC and warfarin groups, respectively), more likely to be men (62% vs. 52% for men and women, respectively) and presented more risk factors for stroke as estimated by CHA_2_DS_2_-VASc score (mean ± standard deviation: 4.1 ± 1.6) are lower than those using warfarin (mean ± standard deviation: 3.5 ± 1.9). After the study population had been weighted using a 5% trimmed IPTW method, all differences of baseline covariates were less than an ASD of 0.1. The two treatment groups were well-balanced with sufficient overlap in the inspection of individual propensity score distributions (Figure S1, Supplementary Materials). The mean age was 71.2 years, and the mean CHA_2_DS_2_-VASc score was 3.9. In weighted cohorts, the proportion of non-rheumatic mitral stenosis was 1.0%, that of mitral regurgitation was 43.7%, and those of other VHDs were 55.3% in the NOAC group. In the warfarin group, those of non-rheumatic mitral stenosis was 3.1%, that of mitral regurgitation was 40.9%, and those of other VHDs were 56.0%.

### 3.2. Clinical Outcomes in Patients with AF and EHRA Type 2 VHDs

**Figure 2:**
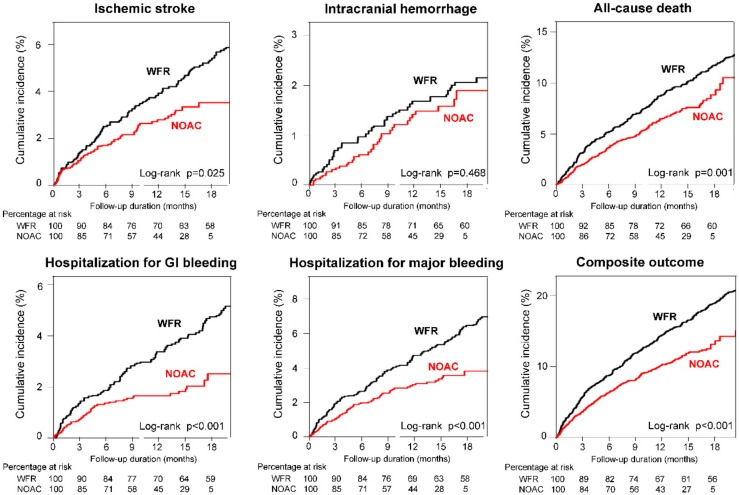
Weighted cumulative incidence curves of 6 clinical outcomes of NOAC versus warfarin. Compared to warfarin, NOACs showed significantly lower incidence rates of ischemic stroke, GI bleeding, major bleeding, and all-cause mortality. NOAC and warfarin groups showed similar incidence rates of intracranial hemorrhage (ICH). Overall, NOACs were associated with a lower rate of the composite outcome. Abbreviations: GI, gastrointestinal; NOAC, non-vitamin K antagonist oral anticoagulant; WFR, warfarin.

**Figure 3:**
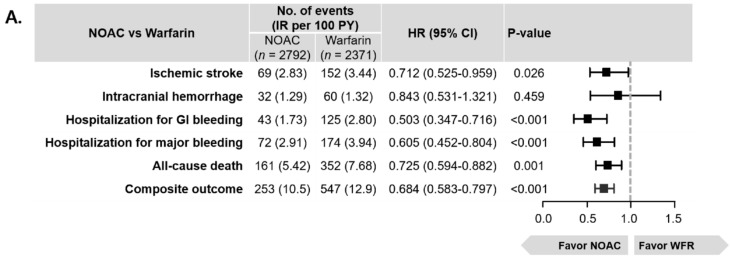

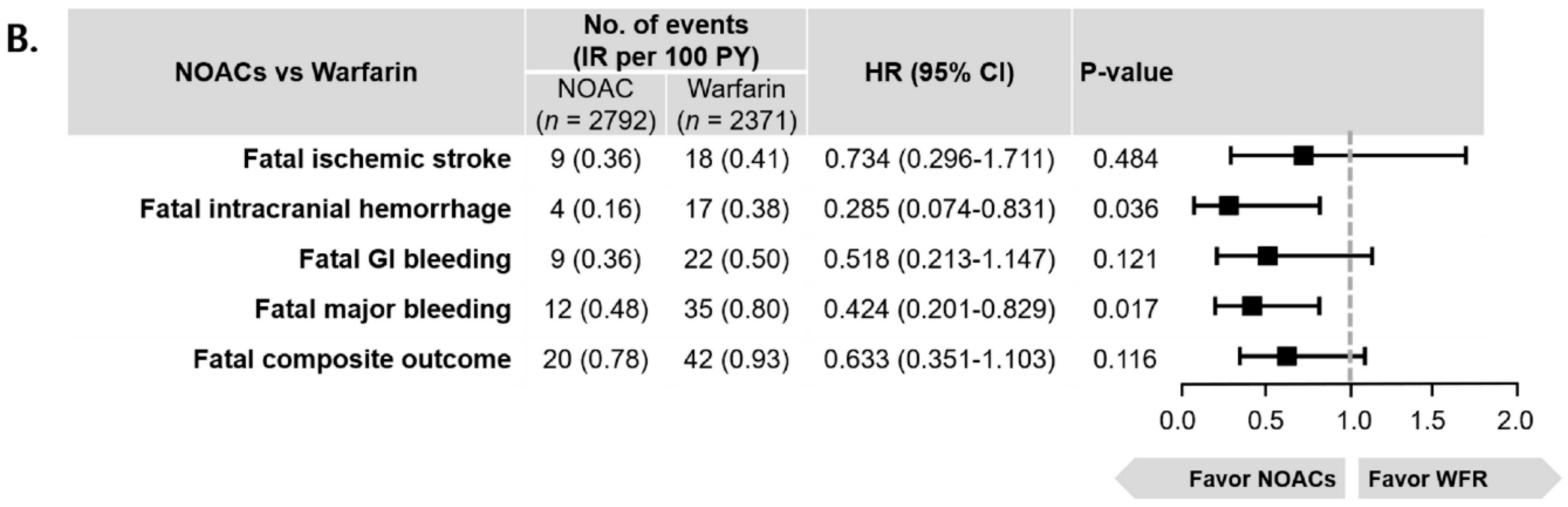
Weighted event numbers, incidence rates, and HRs of 6 clinical outcomes and fatal events in NOAC versus warfarin (reference) in patients with EHRA type 2 VHDs: (**A**) HRs of 6 clinical outcomes; (**B**) HRs of fatal clinical events. Compared to warfarin, NOACs were associated with lower risks of ischemic stroke, GI bleeding, and major bleeding. Although NOAC and warfarin groups showed similar incidence rates of ICH, the NOAC group was associated with a significantly lower risk of fatal ICH compared to warfarin group. Overall, NOACs were associated with a lower risk of the composite outcome (HR: 0.68, 95% CI: 0.58–0.80). Abbreviations: CI, confidence interval; GI, gastrointestinal; HR, hazard ratio; ICH, intracranial hemorrhage; NOAC, non-vitamin K antagonist oral anticoagulant.

Weighted cumulative incidence curves for the 6 clinical outcomes are shown in Figure 2. During the follow-up, weighted incidence rates for ischemic stroke was 2.83 per 100 PY in the NOAC group and 3.44 per 100 PY in the warfarin group (Figure 3, panel A). NOAC use was associated with lower rates of ischemic stroke compared to warfarin: The HR was 0.712 (95% CI: 0.525–0.959) (Figure 3, panel A). 

When focusing on the safety outcomes, ICH was observed at weighted incidence rates of 1.29 per 100 PY for NOAC and 1.32 per 100 PY for warfarin. NOAC showed a nonsignificant trend for a lower risk of ICH when compared to warfarin (HR: 0.843, 95% CI: 0.523–1.321, Figure 3, panel A). Considering hospitalization for GI bleeding and major bleeding, NOAC (HR for GI bleeding: 0.503, 95% CI: 0.347–0.716) significantly reduced the risks compared to warfarin (HR for major bleeding: 0.605, 95% CI: 0.452–0.804) (Figure 3, panel A). Additionally, NOACs were associated with a 27% lower risk of all-cause death (HR: 0.725, 95% CI: 0.594–0.882) and a better composite outcome compared to warfarin (HR: 0.684, 95% CI: 0.583–0.797) (Figure 3, panel A). Concerning fatal events, NOAC (HR of fatal ICH: 0.285, 95% CI: 0.074–0.831) showed a lower risk of fatal ICH and major bleeding than warfarin (HR of fatal major bleeding: 0.424, 95% CI: 0.201–0.829) (Figure 3, panel B).

### 3.3. Sensitivity Analysis

Consistent results were depicted by the sensitivity analysis using a multivariable Cox regression model. NOAC was associated with better outcomes than warfarin except for ICH, with similar HRs for all 6 clinical outcomes as shown using IPTW analysis (Figure S2, Supplementary Materials). Since the treatment choice might be affected by patients’ socioeconomic status, we additionally ascertained outcomes about patients’ income levels. There were no significant differences in the proportion of patients with low income between NOAC and warfarin groups (Table S4, Supplementary Materials). After adjusting for low income, the HRs for 6 clinical outcomes were consistent with the main results (Table S5, Supplementary Materials).

In additional analyses for other confounding factors, the proportions of liver disease and antiplatelets were not significantly different between the two groups. The warfarin group showed a higher proportion of CKD than the NOAC group (6.87% vs. 4.44%, ASD = 0.155; Table S6, Supplementary Materials). After adjusting for these factors, the HR for 6 clinical outcomes were consistent with the main results (Table S7, Supplementary Materials).

Additional sensitivity analyses were performed to redeem the difference of follow-up duration between two groups. The HR trends for 6 clinical outcomes were still consistent compared with the main results (Table S8, Supplementary Materials). In the on-treatment analysis, the results were also consistent with the main analysis (Table S9, Supplementary Materials).

### 3.4. Subgroup Analyses Stratified by Age, Sex, CHA_2_DS_2_-VASc Score, and VHD Types

**Figure 4:**
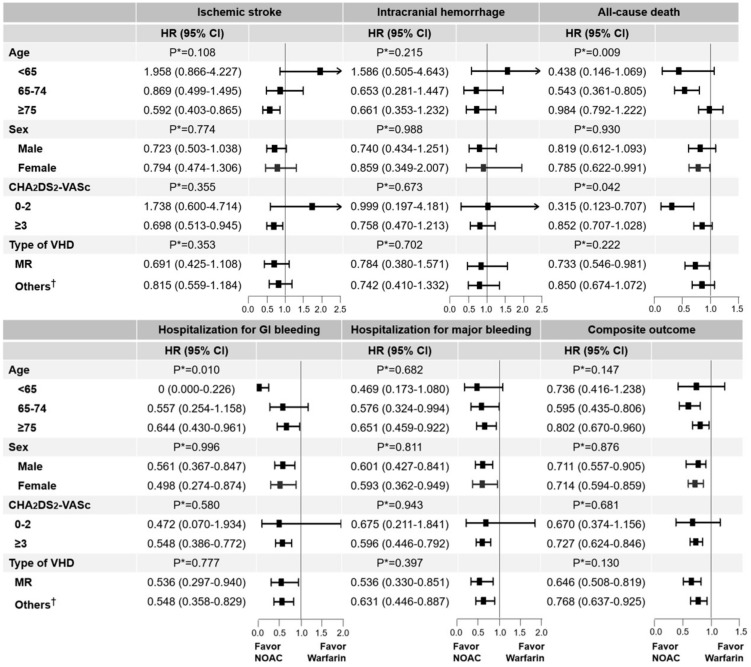
HRs of ischemic stroke, intracranial hemorrhage, all-cause death, GI bleeding, major bleeding, and composite outcome according to various subgroups in NOAC and warfarin (reference). * P for interaction; ^†^ Other valvular disease included AS, AR, TS, TR, PS, and PR. Abbreviations: AR, aortic regurgitation; AS, aortic stenosis; CI, confidence interval; GI, gastrointestinal; HR, hazard ratio; MR, mitral regurgitation; NOAC, non-vitamin K antagonist oral anticoagulant, PR, pulmonary regurgitation; PS, pulmonary stenosis; TR, tricuspid regurgitation; TS, tricuspid stenosis; VHD, valvular heart disease.

The crude incidences of outcomes on NOAC and warfarin by various subgroups are presented in Table S10 (Supplementary Materials), and the adjusted HR for 6 clinical outcomes are shown in Figure 4.

### 3.5. Age (<64, 65–74, and ≥75 Years) and Sex

There were no significant interactions with respect to ischemic stroke, ICH, hospitalization for major bleeding, and the composite outcome between treatment groups and age subgroups, except for hospitalization for GI bleeding and all-cause death. Additionally, no significant interaction was observed between the treatment group and sex of individuals.

### 3.6. CHA_2_DS_2_-VASc Score (0–2 and ≥3)

Except for all-cause death, no interaction was observed between treatment and CHA_2_DS_2_-VASc score. The benefits of NOACs compared to those warfarin were consistent in both CHA_2_DS_2_-VASc scores of 0–2 and ≥3. The group with a CHA_2_DS_2_-VASc score of 0–2 showed a wide CI due to the low rates of the event. A significant interaction for all-cause death was found, whereby the HR showed a beneficial trend for risk in the NOAC group.

### 3.7. Type of VHD (Mitral Regurgitation and Other VHDs)

NOAC use was associated with a risk reduction in all 6 clinical outcomes compared to the use warfarin in both mitral regurgitation and other types of VHD. There was no significant interaction between the treatment and patients’ VHD type.

### 3.8. Comparative Effectiveness and Safety of NOAC versus Warfarin in Patients with EHRA Type 1 VHDs: An Exploratory Analysis

**Figure 5:**
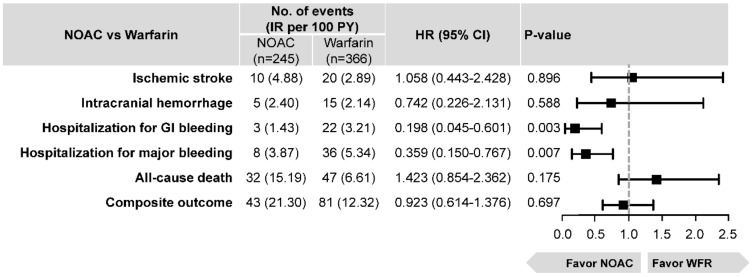
Event numbers, crude incidence rates, and HRs of six clinical outcomes with pooled NOAC versus warfarin (reference) using multivariate Cox regression analysis in patients with EHRA type 1 VHDs. The NOAC group showed a comparable risk of ischemic stroke, ICH, all-cause death, and composite outcome. In hospitalization for GI bleeding and major bleeding, NOAC use was associated with a significantly lower risk compared to the use of warfarin. Abbreviations: CI, confidence interval; EHRA, Evaluated Heartvalves, Rheumatic or Artificial; GI, gastrointestinal; HR, hazard ratio; ICH, intracranial hemorrhage; NOAC, non-vitamin K antagonist oral anticoagulant.

We analyzed patients with EHRA type 1 VHDs, a total number of 611 subjects (warfarin: *n* = 366, NOAC: *n* = 245) in an exploratory analysis. Baseline characteristics and crude incidences are presented in Table S11 (Supplementary Materials) and Figure 5. In the multivariable Cox regression analysis, the NOAC group showed a comparable risk of ischemic stroke, ICH, all-cause death, and composite outcome. In hospitalization for GI bleeding and major bleeding, NOAC use was associated with a significantly lower risk compared to the use of warfarin (Figure 5).

## 4. Discussion

This study is the first Asian population-based study to investigate the effectiveness and safety of NOACs in AF patients with VHDs. The main findings of our study are as follows: (i) NOACs were associated with lower risks of ischemic stroke and all-cause death compared to warfarin; (ii) for safety outcomes, NOACs significantly reduced the risk of hospitalization for GI bleeding and major bleeding and showed a comparable outcome in ICH but a significant reduction in fatal ICH; (iii) overall, NOAC showed a better composite outcome than warfarin in patients with AF and EHRA type 2 VHDs; and (iv) NOAC showed comparable outcomes of 6 clinical outcomes in patients with EHRA type 1 VHDs.

### 4.1. NOACs in Patients with Non-Valvular AF and EHRA Type 2 VHDs

The efficacy and safety of NOACs compared to those of warfarin in patients with non-valvular AF and EHRA type 2 VHDs have been reported in a meta-analysis of previous pivotal clinical trials of NOAC and a few retrospective analyses based on claims databases [10]. In a previous meta-analysis, patients with EHRA type 2 VHDs (total *n* = 13,585 treated with warfarin and NOAC) showed a trend for higher rates of ischemic stroke/systemic embolic events, significantly higher rates of major bleeding and all-cause death than without EHRA type 2 VHDs [10]. Within EHRA type 2 VHD patients, pooled standard-dose NOAC was associated with a lower risk of thromboembolic events (HR: 0.70, 95% CI: 0.58–0.86) and ICH (HR: 0.47, 95% CI: 0.24–0.93) than warfarin, whereas NOAC showed a comparable risk of major bleeding (HR: 0.93, 95% CI: 0.68–1.27). In contrast, NOAC did not reduce the risk of all-cause death (HR: 1.01, 95% CI: 0.90–1.14). There were some differences in the results according to the types of NOAC [10]. Apixaban and higher-dose dabigatran showed a significantly lower risk of SSEE compared to warfarin, whereas higher-dose edoxaban and rivaroxaban showed comparable results. Regarding major bleeding events, rivaroxaban was associated with a higher risk compared to warfarin, whereas other NOACs were not. Nevertheless, the number of each NOAC type was small to find any significant statistical difference.

Noseworthy et al. reported pooled NOAC versus warfarin data in patients with AF and VHDs using claims data in the U.S. [11]. NOAC shows a significantly lower risk for ischemic stroke/systemic embolism (HR: 0.76, 95% CI: 0.59–0.98) and major bleeding (HR: 0.84, 95% CI: 0.72–0.97) compared to warfarin, only in patients with aortic stenosis/insufficiency or mitral regurgitation group. There are no significant differences in patients with non-rheumatic mitral stenosis, rheumatic mitral stenosis, valve repairs, and bioprosthetic valves. Another previous study based in the U.S. claims data analyzed the effectiveness and safety of dabigatran or rivaroxaban versus warfarin in patients with non-valvular AF and VHDs except prosthetic heart valves (dabigatran: *n* = 1979; rivaroxaban: *n* = 2027; and warfarin: *n* = 14,131) [12]. Both dabigatran and rivaroxaban show a comparable risk of stroke and any bleeding compared to warfarin; however, both are associated with the lower risk of all-cause death (dabigatran: An HR of 0.71, 95% CIs of 0.52–0.98; rivaroxaban: An HR of 0.68, 95% CIs of 0.49–0.95).

In our study, incidence rates of clinical events were higher in patients with non-valvular AF and EHRA type 2 VHD than those in general patients with non-valvular AF [14,23] This was consistent with previous observational reports [24,25] and NOACs versus warfarin studies [10,11,12]. The difference in VHD distribution type may lead to slightly different results in the effectiveness and safety of NOACs compared to warfarin among studies [10,11,12]. Indeed, mitral regurgitation has been suggested to have a protective effect for stroke in AF [26,27], and this might be driven by the higher flow patterns in the left atrium [1]. The association of aortic stenosis with stroke has been controversial because potential bidirectional effects in coagulation were observed. Aortic stenosis could increase platelet production [28], and activate coagulation and platelets with turbulent flow through valves [29]. However, there is evidence that aortic stenosis could impair hemostasis with acquired von Willebrand syndrome [30]. In a post hoc analysis of Rivaroxaban-Once Daily, Oral, Direct Factor Xa Inhibition Compared with Vitamin K Antagonism for Prevention of Stroke and Embolism Trial in Atrial Fibrillation (ROCKET AF) trial, patients with aortic stenosis had an increased risk of stroke, systemic embolism, and major bleeding compared to patients with other VHDs or without VHDs [31].

Comparing to the proportion of VHD types, mitral regurgitation incidence in our study was lower compared to that of pooled pivotal clinical trials of NOAC (42% vs. 70–80%) and aortic stenosis was higher (17% vs. 10%). According to Noseworthy et al., patients with aortic stenosis/insufficiency and mitral regurgitation are 96% of the total cohort [11], but a detailed distribution of VHD type was not reported. Beyond these differences from previous studies, in our large Asian-based cohort with AF and EHRA type 2 VHDs, the effectiveness and safety of NOAC were maintained in the patients with AF and EHRA type 2 VHDs.

In pivotal NOAC RCTs, a smaller subgroup of Asian patients compared to non-Asians were included, with some differences reported in outcomes [13,32]. Asians patients with AF are known to have a higher risk of stroke/systemic embolism and major bleeding when using warfarin compared with non-Asian patients with AF. However, Asian patients had a higher risk reduction of efficacy and safety endpoints with NOACs versus warfarin compared with non-Asian patients [33]. In recent real-world studies, the effectiveness and safety of NOACs have also been reported, even with edoxaban [14,15,19]. In line with the Asian data in the broader AF population, the present study shows that NOACs are associated with a lower risk of ischemic stroke and bleeding in Asian patients with AF and VHDs.

### 4.2. NOACs in AF Patients with EHRA Type 1 VHDs (Valvular AF)

Patients with valvular AF including severe rheumatic mitral stenosis and prosthetic heart valves were excluded from the pivotal clinical trial of NOACs [2,3,4,5,6], and the guideline recommended warfarin in these patients [34]. Noseworthy et al. reported the clinical outcomes of NOAC in 74 AF patients with rheumatic mitral stenosis [11], showing no ischemic stroke/systemic embolism events in both NOAC and warfarin groups, with no statistically significant differences for major bleeding. In our cohort, we identified 611 patients with EHRA type 1 VHDs, including rheumatic mitral stenosis and prosthetic heart valves (245 patients treated with NOAC and 366 patients treated with warfarin). NOAC use showed similar results in ischemic stroke, ICH, and all-cause death compared with the use of warfarin; however, NOAC showed better safety with a comparable net clinical benefit compared with warfarin. However, the numbers of patients with EHRA type 1 VHDs in our exploratory analysis were too small to conclude the effectiveness and safety of NOAC compared to those of warfarin in these patients.

### 4.3. The Clinical Implication with Subgroup Analyses

Clinical outcome trends were generally consistent, but there were some significant interactions in age and CHA_2_DS_2_-VASc score subgroups. In the younger age group (less than 65 years old), the protective effects of NOAC on all-cause death and GI bleeding were higher. In the lower-CHA_2_DS_2_-VASc-score group (0 to 2 points), NOAC use showed a 70% lower risk compared with the use of warfarin and those were not different in the higher-CHA_2_DS_2_-VASc-score group. In line with previous studies, we found that mitral regurgitation tends to show better outcomes than other VHDs. 

### 4.4. Study Limitations

Several limitations of the study should be considered. First, diagnoses were defined by ICD-10 codes or prescriptions in studies using claims data, which might cause the inherent limitation of coding errors (over or under coding) or clinical inaccuracy. Although there was no financial incentive for accurate coding of clinical diagnoses in Korea, the definition of AF and comorbidities and clinical outcomes have been validated in several previous studies [9,14,15,19]. Second, echocardiography is needed to diagnose VHD accurately. However, this examination was not reimbursed in Korea until recently, so we could not provide the precise numbers of patients who had echocardiography evaluation during all years. However, the 2018 Korean guidelines of AF management recommended performing echocardiography in patients with newly diagnosed AF for distinguishing valvular AF from non-valvular AF or for examining the presence of heart failure or other structural heart diseases [35]. Thus, contemporary practice means that VHD would be more easily diagnosed with routine echocardiography. Third, confounding factors might still exist despite well-balanced groups using the propensity score weighting method. Although NOACs were reimbursed in patients with a CHA_2_DS_2_-VASc score of ≥2 in Korea, patients’ copay between NOAC and warfarin was still different. However, we found that the proportions of patients with low income were not different between NOAC and warfarin groups and the HRs for 6 clinical outcomes adjusting for low income were consistent with the main results. Fourth, the follow-up period for outcomes was shorter in the NOAC group than in the warfarin group because NOACs have only been used widely since 2015 in Korea. To address this limitation, we performed sensitivity analyses with the restriction of the follow-up period, and the results remained consistent. Fifth, owing to the inherent limitation weakness of the claims database, we could not evaluate the treatment quality of the warfarin group. The lower time in the therapeutic range (TTR) control was reported in the Asian population [36,37], and it could be a possible confounding factor in a favorable result of NOAC regarding ischemic stroke. Finally, patients with prior ischemic stroke, ICH, or GI bleeding were excluded from this study for accurate assessment of clinical outcomes. The results of our study should, therefore, be cautiously interpreted and applied to patients with a previous history of ischemic stroke, ICH, or GI bleeding.

## 5. Conclusions

In this nationwide Asian AF population with EHRA type 2 VHDs, NOAC use was associated with lower risks of ischemic stroke, major bleeding, all-cause death, and the composite outcome compared to the warfarin use.

## Figures and Tables

**Table 1:** Baseline characteristics of patients with EHRA type 2 VHDs between warfarin versus NOACs.

	Propensity Score Weighting					
	Before			After (with 5% Trimming)		
	Warfarin (*n* = 2671)	NOACs (*n* = 3058)	ASD	Warfarin (*n* = 2371)	NOACs (*n* = 2792)	ASD
**Age, years**	66.5 ± 13.2	73.5 ± 9.2	0.620	71.2 ± 9.9	71.2 ± 8.4	0.002
**<65**	39.4	14.9		24.5	21.3	
**65–74**	30.4	34.1		33.6	38.2	
**≥75**	30.2	50.9		41.8	40.5	
**Men**	51.7	61.6	0.200	57.3	56.9	0.006
**CHA_2_DS_2_-VASc score**	3.5 ± 1.9	4.1 ± 1.6	0.340	3.95 ± 1.85	3.94 ± 1.63	0.003
**0–2**	31.5	16.1		22.8	19.8	
**≥3**	68.5	83.9		77.2	80.2	
**Hypertension**	73.9	76.4	0.057	76.7	76.5	0.005
**Diabetes mellitus**	16.5	17.9	0.036	18.4	18.5	0.000
**Dyslipidemia**	36.4	40.1	0.076	40.3	39.5	0.015
**Heart failure**	52.5	49.5	0.059	51.6	51.3	0.005
**Prior MI**	4.3	3.5	0.043	4.2	4.2	0.002
**PAD**	15.5	18.5	0.081	18.3	18.0	0.006
**COPD**	24.6	25.2	0.012	26.2	26.2	0.000
**NOAC dose ^†^**						
**Regular dose**	N.A	36.6	N.A	N.A	41.1	N.A
**Reduced dose**	N.A	63.4	N.A	N.A	58.9	N.A

Values are mean ± standard deviation or %. Other valvular disease included AS, AR, TS, TR, PS, and PR. ^†^ Regular-dose NOACs are 20 mg rivaroxaban once daily, 150 mg dabigatran twice daily, 5 mg apixaban twice daily, and 60 mg edoxaban once daily. Reduced-dose NOACs are 15/10 mg rivaroxaban once daily, 110 mg dabigatran once daily, 2.5 mg apixaban twice daily, and 30 mg edoxaban once daily. Abbreviations: AR, aortic regurgitation; AS, aortic stenosis; ASD, absolute standardized difference; COPD, chronic obstructive pulmonary disease; MI, myocardial infarction; MR, mitral regurgitation; MS, mitral stenosis; PAD, peripheral artery disease; PR, pulmonary regurgitation; PS, pulmonary stenosis; TR, tricuspid regurgitation; TS, tricuspid stenosis.

## Supplementary Materials

The following are available online at https://www.mdpi.com/2077-0383/8/10/1624/s1, Table S1: Definitions of covariates, Table S2: Definitions of clinical outcomes, Table S3: The logistic regression model used to calculate the propensity score, Table S4: The proportion of patients with low income, Table S5: Hazard ratios of 6 clinical outcomes for NOAC versus warfarin after additional adjusting for low income, Table S6: The proportion of patients with chronic kidney disease (CKD), liver disease, and antiplatelets, Table S7: Hazard ratios of 6 clinical outcomes for NOAC versus warfarin after additional adjusting for CKD, liver disease, and antiplatelets, Table S8: Sensitivity analysis restricting the follow-up period to 6 months or 1 year, Table S9: Sensitivity analysis: Hazard ratios of 6 clinical outcomes for pooled NOAC versus warfarin by on-treatment analysis, Table S10: Number of events, crude event rates according to various subgroups in patients with EHRA type 2 VHDs, Table S11: Baseline characteristics of patients with EHRA type 1 VHDs, Figure S1: Distribution of propensity scores in pooled NOAC and warfarin groups before and after weighting, Figure S2: Hazard ratios of 6 clinical outcomes with pooled NOAC versus warfarin using multivariate Cox regression analysis in patients with EHRA type 2 VHDs.
