# Supplementary material for: Non-Vitamin K Antagonist Oral Anticoagulants in Patients with Atrial Fibrillation and Valvular Heart Disease
Source: J Clin Med. 2019 Oct 4;8(10):1624. doi: 10.3390/jcm8101624 (PMC6832302; doi:10.3390/jcm8101624)

SUPPLEMENTAL MATERIAL

Table S1. Definitions of covariates

Diagnosis	ICD-10-CM code and definition	Diagnostic definition
**Atrial fibrillation**	I480-484, I489	Admission or outpatient department≥1
**EHRA type 2 valvular heart disease**		
**Mitral valve disease**	I340-I342	
**Aortic valve disease**	I350-I352, I060-I062	Admission≥1
**Tricuspid valve disease**	I360-I362, I070-I072	
**Pulmonary valve disease**	I370-I372	
**EHRA type 1 valvular heart disease**		
**Rheumatic mitral stenosis**	I050, I052, I059	Admission or outpatient department≥1
**Prosthetic heart valves**	Z952-Z954	
**Pulmonary embolism**	I26	Admission≥1
**Deep vein thrombosis**	I802	Admission≥1
**Received joint replacement operation**	N0711, N1711, N1721, N2070, N3710, N3721, N3717, N3720, N2072, N2077, N3722, N3727	Admission≥1
**End stage renal disease**	N185, Z49	Dialysis≥2
**Ischemic stroke**	I63, I64	Admission≥1 and brain imaging (CT or MRI) ≥1
**Intracranial hemorrhage**	I60-62	Admission≥1 or RBC transfusion≥1
**Hospitalization for GI bleeding**	K22.6, K25.0, K25.2, K25.4, K25.6, K26.0, K26.2, K26.4, K26.6, K27.0, K27.2, K27.4, K27.6, K28.0, K28.2, K28.4, K28.6, K29.0, K62.5, K92.0, K92.1, K92.2	Admission≥1 and RBC transfusion≥1

**Hypertension**	I10-I13, I15; and minimum 1 prescription of anti-hypertensive drug (thiazide, loop diuretics, aldosterone antagonist, alpha-/beta-blocker, calcium-channel blocker, angiotensin-converting enzyme inhibitor, angiotensin II receptor blocker).	Admission≥1 or outpatient department≥2
**Diabetes mellitus**	E11-E14; and minimum 1 prescription of anti-diabetic drugs (sulfonylureas, metformin, meglitinides, thiazolidinediones, dipeptidyl peptidase-4 inhibitors, α-glucosidase inhibitors, and insulin).	Admission≥1 or outpatient department≥2
**Dyslipidemia**	E78	Admission or outpatient department≥1
**Heart failure**	I50	Admission or outpatient department≥1
**Vascular disease**		
**Prior MI**	I21, I22	Admission or outpatient department≥1
**PAD**	I70, I73	Admission or outpatient department≥2
**Chronic kidney disease**	N18	Admission or outpatient department≥1
**Chronic hepatitis**	B15-19	Admission or outpatient department≥1
**Liver cirrhosis**	K70-77	Admission or outpatient department≥1
**COPD**	J41-44	Admission or outpatient department≥1

Abbreviation: COPD, chronic obstructive pulmonary disease; GI, gastrointestinal; MI, myocardial infarction; PAD, peripheral artery disease.

Table S2. Definitions of clinical outcomes

Clinical outcomes	ICD-10-CM code and definition	Diagnostic definition
**Ischemic stroke**	I63, I64	Admission≥1 and brain imaging (CT or MRI) ≥1
**Intracranial hemorrhage**	I60-62	Admission≥1 or RBC transfusion≥1
**Hospitalization for GI bleeding**	K22.6, K25.0, K25.2, K25.4, K25.6, K26.0, K26.2, K26.4, K26.6, K27.0, K27.2, K27.4, K27.6, K28.0, K28.2, K28.4, K28.6, K29.0, K62.5, K92.0, K92.1, K92.2	Admission≥1 and RBC transfusion≥1
**Hospitalization for major bleeding**	Intracranial hemorrhage or gastrointestinal bleeding	ICH, admission≥1 or RBC transfusion≥1<br>GI bleeding, admission≥1 and RBC transfusion≥1
**Composite outcome**	Ischemic stroke, intracranial hemorrhage, gastrointestinal bleeding or all-cause death	Ischemic stroke, Admission≥1 and brain imaging (CT or MRI) ≥1<br>ICH, admission≥1 or RBC transfusion≥1<br>GI bleeding, admission≥1 and RBC transfusion≥1

Abbreviation: GI, gastrointestinal; ICH, intracranial hemorrhage.

Table S3. The logistic regression model used to calculate the propensity score

	Coefficient	SE	OR (95% CI)	P value
**Age (per year)**	0.060	0.003	1.061 (1.057-1.066)	<0.001
**Sex (male)**	-0.113	0.032	0.645 (0.601-0.693)	<0.001
**CHA_2DS_2-VASc score (per point)**	-0.044	0.031	0.850 (0.824-0.876)	0.149
**Hypertension**	0.012	0.034	1.031 (0.964-1.103)	0.731
**Diabetes mellitus**	-0.016	0.039	1.079 (0.996-1.169)	0.675
**Dyslipidemia**	-0.050	0.029	1.129 (1.061-1.201)	0.089
**Congestive heart failure**	0.070	0.033	0.742 (0.692-0.796)	0.034
**Prior MI**	0.185	0.073	0.764 (0.656-0.889)	0.011
**COPD**	0.062	0.032	0.863 (0.805-0.926)	0.056
**PAD**	-0.028	0.041	1.265 (1.164-1.375)	0.490

Abbreviation: CI, confidence interval; COPD, chronic obstructive pulmonary disease; MI, myocardial infarction; OR, odds ratio; PAD, peripheral artery disease; SE, standard error of coefficient.

Table S4. The proportion of patients with low income

	Before IPTW			After IPTW with 5% trimming		
	**Warfarin**	**NOACs**	**ASD**	**Warfarin**	**NOACs**	**ASD**
**Low income (%)**	25.6	25.3	0.008	24.6	24.8	0.003

Abbreviation: ASD, absolute standardized difference; IPTW, inverse probability of treatment weighting; NOAC, non-vitamin K antagonist oral anticoagulant.

Table S5. Hazard ratios of 6 clinical outcomes for NOAC versus warfarin after additional adjusting for low income

NOAC vs. Warfarin	HR (95% CI)	p-value
**Ischemic stroke**	0.712 (0.527-0.962)	0.026
**Intracranial hemorrhage**	0.842 (0.535-1.326)	0.459
**Hospitalization for GI bleeding**	0.503 (0.351-0.722)	<0.001
**Hospitalization for major bleeding**	0.605 (0.454-0.807)	<0.001
**All-cause death**	0.725 (0.595-0.883)	0.001
**Composite outcome**	0.683 (0.584-0.798)	<0.001

Abbreviation: CI, confidence interval; GI, gastrointestinal; HR, hazard ratio; NOAC, non-vitamin K antagonist oral anticoagulant.

Table S6. The proportion of patients with CKD, liver disease, and antiplatelets

	After IPTW with 5% trimming		
	**Warfarin (n=2,371)**	**NOACs (n=2,792)**	**ASD**
**CKD (%)**	163 (6.9%)	124 (4.4%)	0.155
**Chronic hepatitis (%)**	180 (7.6%)	221 (7.9%)	0.026
**Liver cirrhosis (%)**	41 (1.7%)	38 (1.4%)	0.009
**Antiplatelets (%)**	1802 (76.0%)	2201 (78.9%)	0.056

Abbreviation: ASD, absolute standardized difference; CKD, chronic kidney disease; IPTW, inverse probability of treatment weighting; NOAC, non-vitamin K antagonist oral anticoagulant.

Table S7. Hazard ratios of 6 clinical outcomes for NOAC versus warfarin after additional adjusting for CKD, liver disease, and antiplatelets

NOAC vs. Warfarin	CKD and liver disease		Antiplatelets		CKD, liver disease, and antiplatelets	
	HR (95% CI)	p-value	HR (95% CI)	p-value	HR (95% CI)	p-value
Ischemic stroke	0.743 (0.550-1.005)	0.054	0.709 (0.525-0.957)	0.025	0.741 (0.548-1.002)	0.052
Intracranial hemorrhage	0.901 (0.570-1.423)	0.654	0.842 (0.535-1.326)	0.459	0.901 (0.570-1.423)	0.654
Hospitalization for GI bleeding	0.544 (0.379-0.782)	0.001	0.503 (0.351-0.722)	<0.001	0.545 (0.379-0.783)	0.001
Hospitalization for major bleeding	0.649 (0.486-0.867)	0.004	0.605 (0.454-0.807)	0.001	0.650 (0.487-0.868)	0.004
All-cause death	0.761 (0.624-0.928)	0.007	0.724 (0.594-0.882)	0.001	0.760 (0.623-0.927)	0.007
Composite outcome	0.717 (0.613-0.839)	<0.001	0.680 (0.582-0.796)	<0.001	0.716 (0.612-0.838)	<0.001

Abbreviation: CI, confidence interval; CKD, chronic kidney disease; GI, gastrointestinal; HR, hazard ratio; NOAC, non-vitamin K antagonist oral anticoagulant.

Table S8. Sensitivity analysis restricting the follow-up period to 6-month or 1-year

NOAC vs. Warfarin	HR (95% CI)	P-value
**6-month follow-up**		
**Ischemic stroke**	0.690 (0.462-1.030)	0.069
**Intracranial hemorrhage**	0.614 (0.316-1.192)	0.149
**Hospitalization for GI bleeding**	0.718 (0.456-1.132)	0.153
**Hospitalization for major bleeding**	0.726 (0.496-1.062)	0.098
**All-cause death**	0.692 (0.527-0.911)	0.008
**Composite outcome**	0.710 (0.576-0.875)	0.001
**1-year follow-up**		
**Ischemic stroke**	0.716 (0.513-0.999)	0.049
**Intracranial hemorrhage**	0.826 (0.504-1.353)	0.447
**Hospitalization for GI bleeding**	0.524 (0.354-0.777)	0.001
**Hospitalization for major bleeding**	0.653 (0.478-0.892)	0.007
**All-cause death**	0.720 (0.578-0.897)	0.003
**Composite outcome**	0.696 (0.586-0.826)	<0.001

Abbreviation: CI, confidence interval; GI, gastrointestinal; HR, hazard ratio; NOAC, non-vitamin K antagonist oral anticoagulant.

Table S9. Sensitivity analysis: Hazard ratios of 6 clinical outcomes for pooled NOAC versus warfarin by on-treatment analysis

NOAC vs. Warfarin	HR (95% CI)	P-value
**Ischemic stroke**	0.625 (0.443-0.882)	0.007
**Intracranial hemorrhage**	0.773 (0.468-1.277)	0.314
**Hospitalization for GI bleeding**	0.497 (0.330-0.748)	<0.001
**Hospitalization for major bleeding**	0.582 (0.422-0.803)	0.001
**All-cause death**	0.705 (0.544-0.912)	0.007
**Composite outcome**	0.669 (0.555-0.807)	<0.001

Abbreviation: CI, confidence interval; GI, gastrointestinal; HR, hazard ratio; NOAC, non-vitamin K antagonist oral anticoagulant.

Table S10. Number of events, crude event rates according to various subgroups in patients with EHRA type 2 VHD

Subgroup category	Treatment group	Total number	Ischemic stroke		ICH		Hospitalization for GI bleeding		Hospitalization for major bleeding		All-cause death		Composite outcome*	
			Events	IR†	Events	IR†	Events	IR†	Events	IR†	Events	IR†	Events	IR†
**Age (years)**														
**<65**	**Warfarin**	1052	24	1.2	11	0.5	26	1.3	36	1.8	39	1.9	79	4.0
	**NOACs**	457	11	2.8	6	1.5	0	0	6	1.5	5	1.2	18	4.6
**65-74**	**Warfarin**	813	44	2.9	22	1.4	29	1.9	51	3.3	99	6.3	157	10.6
	**NOACs**	1042	24	2.5	10	1.1	11	1.1	20	2.1	37	3.9	66	7.0
**≥75**	**Warfarin**	806	73	5.2	26	1.8	63	4.5	81	5.8	198	13.4	286	21.6
	**NOACs**	1559	50	3.7	20	1.4	46	3.4	62	4.5	189	13.6	257	19.3
**Sex**														
**Male**	**Warfarin**	1382	83	3.3	39	1.5	71	2.8	104	4.1	185	7.1	298	12.2
	**NOACs**	1884	59	3.5	27	1.6	39	2.3	61	3.6	147	8.6	227	13.7
**Female**	**Warfarin**	1289	58	2.4	20	0.8	47	1.9	64	2.7	151	6.1	224	9.5
	**NOACs**	1174	26	2.5	9	0.9	18	1.7	27	2.6	84	8.1	114	11.3
**CHA_2DS_2-VASc**														
**0-2**	**Warfarin**	840	16	1.1	8	0.5	12	0.8	19	1.3	34	2.3	59	4.0
	**NOACs**	492	7	1.8	3	0.7	2	0.5	5	1.2	7	1.7	19	4.8
**≥3**	**Warfarin**	1831	125	3.6	51	1.4	106	3.0	149	4.3	302	8.3	463	13.9
	**NOACs**	2566	78	3.4	33	1.4	55	2.4	83	3.6	224	9.5	322	14.1

**Type of valvular heart disease**														
**MR**	**Warfarin**	1134	60	2.8	26	1.2	44	2.0	65	3.0	141	6.4	215	10.4
	**NOACs**	1278	30	2.6	14	1.2	19	1.6	28	2.4	82	6.9	119	10.4
**Others** ¥	**Warfarin**	1537	81	2.9	33	1.2	74	2.6	103	3.7	195	6.7	307	11.4
	**NOACs**	1780	55	3.6	22	1.4	38	2.4	60	3.9	149	9.5	222	14.7

*Composite outcome, ischemic stroke + ICH + hospitalization for GI bleeding + all-cause death

†Incidence rate, per 100 person-years

¥ Other valvular disease included AS, AR, TS, TR, PS, and PR.

Abbreviation: AR, aortic regurgitation; AS, aortic stenosis; GI, gastrointestinal; ICH, intracranial hemorrhage; MR, mitral regurgitation; NOAC, non-vitamin K antagonist oral anticoagulant; PR, pulmonary regurgitation; PS, pulmonary stenosis; TR, tricuspid regurgitation; TS, tricuspid stenosis.

Table S11. Baseline characteristics of patients with EHRA type 1 VHD

	Warfarin (n=366)	NOACs (n=245)	P value
**Age, years**	67.2±12.4	74.5±9.0	< 0.001
**<65**	34.7	10.6	
**65-74**	33.3	33.1	
**≥75**	32.0	56.3	
**Men**	47.0	55.1	0.050
**CHA_2DS_2-VASc score**	3.7±1.9	4.4±1.6	< 0.001
**0-2**	27.1	10.6	
**≥3**	73.0	89.4	
**Hypertension**	73.8	77.1	0.345
**Diabetes mellitus**	21.6	22.9	0.710
**Dyslipidemia**	47.5	58.8	0.006
**Heart failure**	56.3	49.0	0.076
**Prior MI**	11.2	13.5	0.400
**PAD**	12.3	17.1	0.093
**COPD**	27.3	26.1	0.743
**NOAC dose†**			
**Regular dose**	-	28.2	-
**Reduced dose**	-	71.8	-

Values are mean ± standard deviation or %.

†Regular dose NOACs are 20 mg rivaroxaban once daily, 150 mg dabigatran twice daily, 5 mg apixaban twice daily, and 60 mg edoxaban once daily. Reduced dose NOACs are 15/10 mg rivaroxaban once daily, 110 mg dabigatran once daily, 2.5 mg apixaban twice daily, and 30 mg edoxaban once daily.

Abbreviation: ASD, absolute standardized difference; COPD, chronic obstructive pulmonary disease; EHRA, Evaluated Heartvalves, Rheumatic or Artificial; MI, myocardial infarction; NOAC, non-vitamin K antagonist oral anticoagulant; PAD, peripheral artery disease; VHD, valvular heart disease.

Figure S1. Distribution of propensity scores in pooled NOAC and warfarin groups before and after weighting

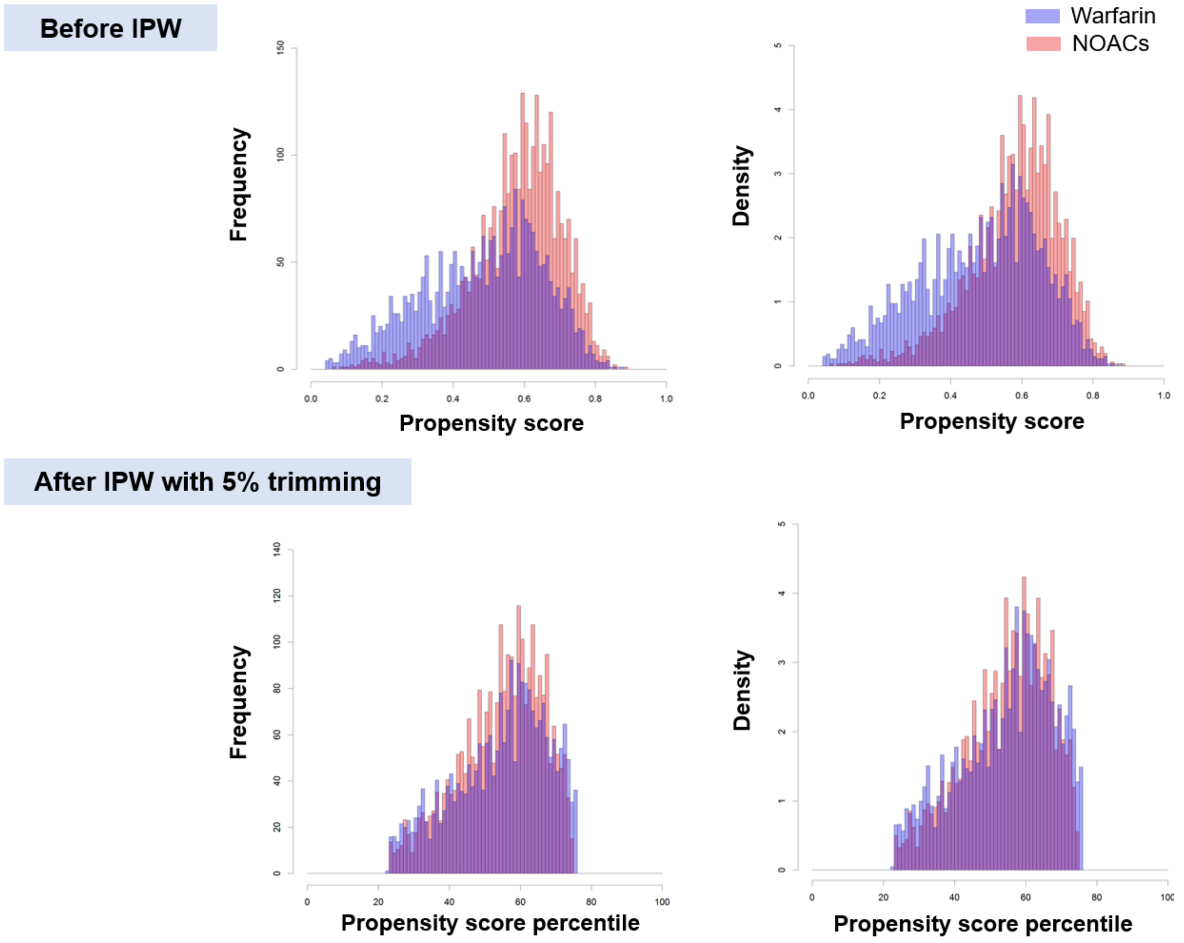

Figure S2. Hazard ratios of six clinical outcomes with pooled NOAC versus warfarin using multivariate Cox regression analysis in patients with EHRA type 2 VHD

NOAC vs Warfarin	HR (95% CI)	P-value
Ischemic stroke	0.752 (0.560-1.007)	0.056
Intracranial hemorrhage	0.767 (0.487-1.200)	0.246
Hospitalization for GI bleeding	0.548 (0.389-0.765)	<0.001
Hospitalization for major bleeding	0.601 (0.455-0.791)	<0.001
All-cause death	0.803 (0.669-0.962)	0.017
Composite outcome	0.718 (0.620-0.831)	<0.001

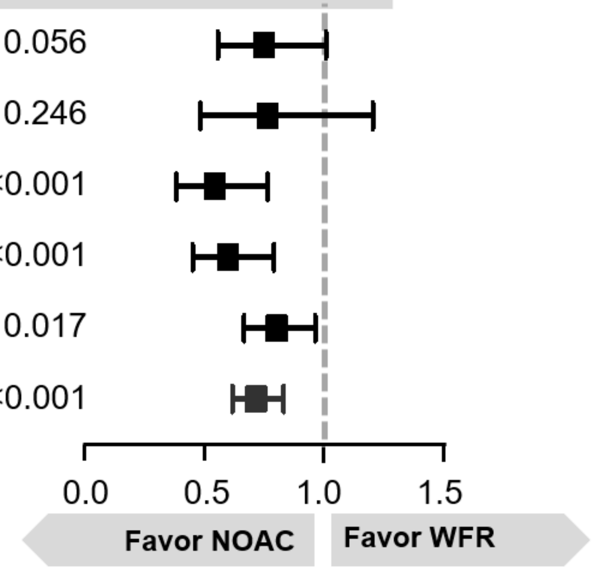

Supplement: Supplementary file 1 [file jcm-08-01624-s001.pdf]
